# Deep learning reconstruction in biparametric prostate MRI: Impact on qualitative and radiomics analyses

**DOI:** 10.1016/j.redii.2025.100059

**Published:** 2025-05-22

**Authors:** Jérémy Dana, Evan McNabb, Juan Castro, Ibtisam Al-Qanoobi, Yoshie Omiya, Kenny Ah-Lan, Véronique Fortier, Giovanni Artho, Caroline Reinhold, Simon Gauvin

**Affiliations:** a McGill University, Department of Diagnostic Radiology, Montréal, Canada; b Research Institute of the McGill University Health Centre, Augmented Intelligence & Precision Health Laboratory (AIPHL), Montréal, Canada; c Université de Strasbourg, Institut Hospitalo-Universitaire (IHU) Strasbourg, Strasbourg, France; d Université de Strasbourg, Inserm U1110, Institute for Translational Medicine and Liver Disease, Strasbourg, France; e McGill University Health Centre, Medical Imaging, Montréal, Canada

**Keywords:** Deep learning reconstruction, Prostate MRI, Qualitative analysis, Radiomics

## Abstract

**Objective:**

To assess the impact of a commercially available deep learning reconstruction (DLR) algorithm on qualitative and radiomics analyses in prostate MRI.

**Methods:**

This single-centre retrospective cohort included 25 consecutive patients who underwent a prostate MRI (1.5 T) in 2022. T2-weighted (T2WI), diffusion-weighted (DWI; b = 50, 1000, extrapolated 2000 s/mm^2^) and apparent diffusion coefficient (ADC) images were reconstructed using DLR and standard (non-DLR) techniques. The two sets were mixed and blind-reviewed independently by six radiologists. Images were qualitatively scored according to PI-QUAL score, overall image quality, diagnostic confidence, anatomical conspicuity, artifact, and noise. Transition and peripheral zones were segmented and radiomics features extracted from region-of-interests using Pyradiomics package. Qualitative criteria and radiomics were compared using a pairwise Wilcoxon signed-rank test.

**Results:**

PI-QUAL score was not significantly different (*p* = 0.32). Overall image quality was not significantly different (*p* = 0.21 on T2WI and 0.56 on DWI/ADC). Noise was lower on DLR images for T2WI (*p* < 0.01) and DWI/ADC (*p* = 0.04). Diagnostic confidence in excluding clinically significant cancer (PI-RADS ≥ 3) in the transition zone was lower with DLR images (p = 0.02). In the transition zone, 89/93 (96 %) of the radiomics features were significantly different between non-DLR and DLR images on T2WI, 68/93 (73 %) on DWI b-2000 s/mm^2^, and 55/93 (59 %) on ADC images. In the peripheral zone, 91/93 (98 %) were significantly different on T2WI, 50/93 (54 %) on DWI b-2000 s/mm^2^, and 70/93 (75 %) on ADC images.

**Conclusion:**

Radiomics features were significantly different on DLR images which should encourage caution for clinical and research purposes. DLR algorithm decreases noise while preserving overall image quality.

## Highlights

• Overall image quality was not significantly improved using DLR on prostate MRI exams.
• Most radiomics features were significantly different between non-DLR and DLR images.
• Classifiers from MRI radiomics features must be aware of the reconstruction method.

## 1 Introduction

Worldwide, prostate cancer is the second most common cancer diagnosed in men (GLOBOCAN database [1]). A priority in the management of men with prostate cancer is the ability to accurately assess the presence of clinically significant cancers, to accurately assess the extent of disease at diagnosis, and to characterize the risk of future progression, thereby avoiding unnecessary overtreatment in men at low risk for progression, and undertreatment that may contribute to treatment failures, especially for patients opting for active surveillance. Magnetic resonance imaging (MRI) offers reliable visualization of potentially significant prostate cancers and has been shown to better select patients for biopsy and to facilitate direct targeting of lesions during biopsy [2]. MRI also provides information for staging tumour extent and monitoring treatment response.

The recent increased use of prostate MRI examinations coupled with the prolonged scanning times for prostate MRI, has become a challenge, particularly in the current context of healthcare system constraints. Accelerating MRI acquisitions has been an active area of research for many years. The major developments that have contributed to faster imaging are parallel imaging and compressed sensing [3,4]. More recently, deep learning reconstruction (DLR) has emerged as a technology to shorten scanning acquisition time and boost signal-to-noise ratio (SNR). However, although the image quality appears improved to the naked eye, its impact on objective criteria such as anatomical conspicuity or MR-related artifacts remains unclear [5], [6], [7]. The same applies to its impact on artifacts with inconsistent reports in the literature [8,9].

DLR directly operates on the reconstruction of k-space and not on the MRI images. Therefore, this type of reconstruction is likely to impact quantitative analysis, including radiomics and deep learning features. This is particularly concerning as more and more machine learning models relying on these quantitative features are being developed. The objective of these models encompasses various clinical tasks, from cancer detection to characterization [10], [11], [12]. If DLR does indeed impact quantitative image analysis, models built using standard non-DLR images may completely fail when applied to images acquired with DLR and vice versa. This may also vary depending on the DLR factor used, markedly limiting generalizability at same site over time and between sites. Moreover, given the need for large data sets for model building and validation, users and researchers will need to carefully assess MRI data sets for the introduction of DL reconstruction algorithms. The extent of this impact remains unknown [9]. There is a paucity of data on the impact of deep learning reconstruction on radiomics features in computed tomography [13] and none in MRI.

We hypothesise that (i) the image quality and MR-related artifacts will not significantly differ between the DLR and non-DLR images, but that (ii) the radiomics features extracted from DLR and non-DLR images will significantly differ between the two reconstruction techniques, which can potentially impact radiomics-based models trained on non-DLR images.

The objective of this study is to assess the impact of a commercially available DLR algorithm on prostate MRI images including diagnostic confidence, image quality, and severity of imaging artifacts and radiomics analysis, compared to current standard non-DLR image reconstruction techniques.

## 2 Material and methods

### 2.1 Patient selection

**Fig. 1:**
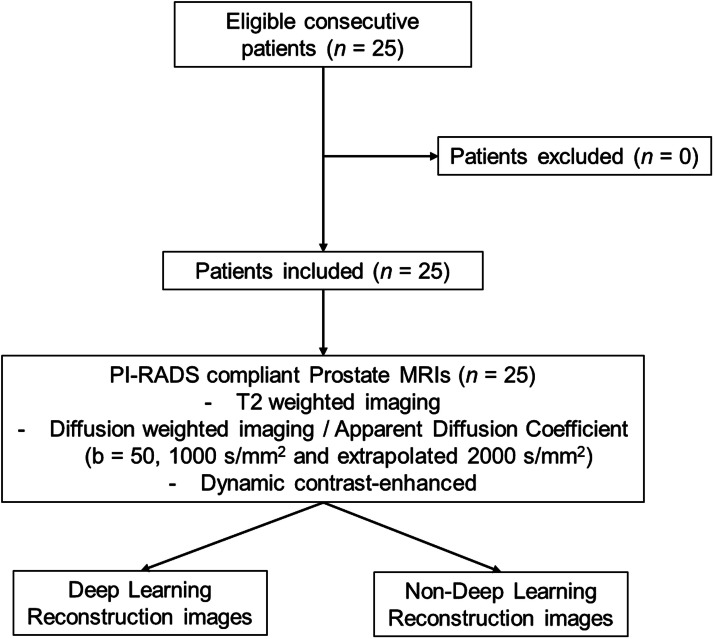
Study is to assess the impact of a commercially available deep learning reconstruction algorithm on prostate MRI: patient selection.

Twenty-five adult male patients who consecutively underwent prostate MRI with both standard non-DLR and DLR algorithms between October and November 2022 were included in this study (Fig. 1).

### 2.2 Imaging protocol

All prostate MRIs were performed on a 1.5 T system (Artist, GE Healthcare, Chicago, USA) equipped with a 30-channel phased-array coil. The imaging protocol included T2-weighted imaging, diffusion-weighted imaging (b-values = 50 and 1000 s/mm^2^), and diffusion kurtosis imaging (b-value = 2000 s/mm^2^). An apparent diffusion coefficient (ADC) map was calculated from the DWI sequence using the two lowest b-values. An extrapolated high b-value (b = 2000 s/mm^2^) was also generated in addition to the separately acquired diffusion kurtosis imaging with a b-value of 2000 s/mm^2^. The T2WI, DWI (b-values = 50, 1000 and an extrapolated 2000 s/mm^2^), and ADC images were reconstructed using both DLR (AIR^TM^ Recon DL) and the standard non-DLR reconstruction techniques from the same acquired sets of images. All images with DLR applied used the highest setting available in the software settings and was kept consistent for the duration of the study. Full acquisition parameters are available in the supplemental material (Table S1). Images were obtained in accordance with the PI-RADS v2.1 imaging standards published by the American College of Radiology [14]. All images were subsequently de-identified and then uploaded on a research picture archiving and computer system (PACS) for review.

### 2.3 Endpoint and reference standard

The composite endpoint to assess the impact of the commercially available DLR algorithm on prostate MRI images included PI-QUAL score, diagnostic confidence in excluding clinically significant cancer (PI-RADS ≥ 3), specific quality and artifacts metrics, and radiomics features (detailed in Radiomics analysis section).

### 2.4 Image quality analysis

To standardize image evaluation across readers, a slide presentation was generated providing detailed definitions and visual examples of prostate MRI image quality metrics according to the published literature. The definitions and imaging examples presented the full spectrum of image quality criteria on a Likert scale from 1 to 5. Six independent board-certified radiologists (three fellowship-trained radiologists [senior] with 5 (XX), 6 (XX) and 12 (XX) years of experience, respectively, and three radiology fellows (XX, XX and XX) [junior], all subspecialized in abdominal and pelvic imaging) completed a training session. The training session consisted of analysing MRI images from 16 patients (scanned on the same system without DLR between September 2021 and October 2022 due to MR-conditional pacemakers) and rating the image quality using the above Likert scale for various image quality metrics (Supplemental material, Table S2). In addition, the readers rated the lesions detected according to PI-RADS v2.1. The results of the training session were collectively reviewed, and clarification and feedback were provided. After completing the training session, the independent readers reviewed the 25 patient MRI examinations that were presented in a random order of DLR vs non-DLR on a radiology-dedicated research PACS station. The readers were blinded to the patient information, to any laboratory or histopathological analysis, sequences labels and to other information related to the MRI sequences and reconstruction technique used. During the first reading session, the readers were asked to review T2WI and DWI (b = 50, 1000, extrapolated 2000 s/mm^2^ and ADC as a whole) using a standardized reading spreadsheet (Table S2) with either standard reconstruction technique or DLR, presented in a random order. During the second session, the 25 MRI studies were again reviewed using either standard non-DLR reconstruction technique or DLR, ensuring that if a study was initially presented during session #1 with non-DLR reconstruction technique, it was presented in session #2 with DLR and vice versa. To minimize the risk of recall bias from the readers, the two sessions took place at least one month apart.

The final score for each criterion consisted of the agreement of at least three of the six readers including at least one senior reader. Otherwise, a consensus was reached with the additional opinion of a senior subspecialty-trained abdominal radiologist with 12 years of experience (XX), who was also blinded to the same information.

### 2.5 Radiomics analysis

Segmentations of the transition/central zone and peripheral zone were manually drawn slice-by-slice on image volumes from three different sequences, in the axial-oblique plane using 3D Slicer [15]: T2WI, and both DWI (b = 50, 1000, and extrapolated 2000 s/mm^2^) and ADC images, by a junior board-certified radiologist with 1 year of experience (XX).

Pyradiomics, an open-source python package compliant with the Image Biomarker Standardisation Initiative, was used to extract 93 radiomics features from each segmentations on a slice-by-slice basis for each of the images list above [16], [17]. All of the feature classes available in Pyradiomics were extracted, which included the following: first orders statistics (18), gray level co-occurrence matrix (GLCM) (24), gray level run length matrix (GLRLM) (14), gray level size zone matrix (GLSZM) (16), gray level dependence matrix (GLDM) (16), and neighbouring gray tone difference matrix (NGTDM) (5). No feature selection or image filtering was performed as this study aimed to exhaustively study the impact of DLR on all radiomics features. Finally, the derived features for each slice were grouped according to anatomical zone and image type and subsequently used in pairwise analysis (described below) between standard reconstruction and DLR.

### 2.6 Statistical analysis

PI-QUAL score, diagnostic confidence, and quality/artifacts metrics were compared between both reading sessions using the pairwise Wilcoxon signed-rank test [18]. Inter-reader agreement was calculated using the Fleiss Kappa [19]. The κ statistic was interpreted as follows: 0.0, poor agreement; 0.01–0.2, slight agreement; 0.21–0.40, fair agreement; 0.41–0.60, moderate agreement; 0.61 – 0.80, substantial agreement; and 0.81–1.00, almost perfect agreement. All 93 radiomics features were compared using a pairwise Wilcoxon signed-rank test [18] grouped by anatomical zone and image type. Multiple comparisons adjustments were performed within each feature class using a false discovery rate of no more than 5 %. The number of comparisons made was equal to the number of features in each class (range: 5–24). The statistical analysis was performed using MATLAB (Release R2023a, The Mathworks Inc., Natwick, MA).

## 3 Results

### 3.1 Population

Patients' charactacteristics are reported in Table 1. Eleven patients (11/25 – 44 %) underwent surgical resection or targeted biopsies. Seven patients had pathologically-proven prostate cancer with the most common group consisting of International Society of Urological Pathology (ISUP) grade group 2.

**Table 1:** Study is to assess the impact of a commercially available deep learning reconstruction algorithm on prostate MRI: patient demographic. Note: data are numbers of patients, with percentage in parentheses.

Age (years), median [range]	67 [65–75]
Prostate-specific antigen (ng/mL), median [range]	6.1 [4.5–11.8]
Pathology available, *n* (%)	11/25 (44 %)
Prostate cancer, *n* (%)	7/11 (64 %)
Grade according to International Society of Urological Pathology, *n* (%)	
Grade 1	1 (14 %)
Grade 2	3 (44 %)
Grade 3	1 (14 %)
Grade 4	1 (14 %)
Grade 5	1 (14 %)

### 3.2 Image quality analysis

**Fig. 2:**
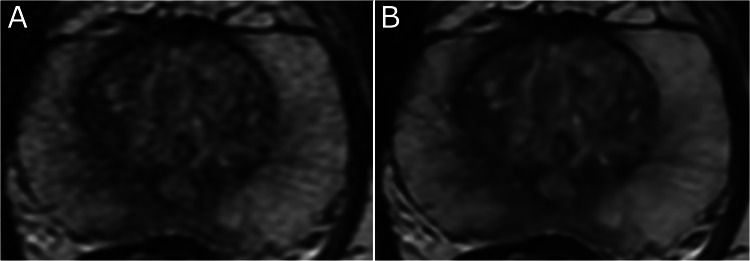
Study is to assess the impact of a commercially available DLR algorithm on prostate MRI: noise on axial T2-weighted images of the prostate, scored 4/5 on standard (non-deep learning reconstructed) images (A), and 5/5 on deep learning reconstructed images (B).

Quality of DLR images was superior to that of non-DLR images only for noise assessment for both T2WI (*p* < 0.01) and DWI/ADC (*p* = 0.04) sequences (Table 2 - Fig. 2). Conversely, motion artifacts were rated more severely on DLR images for T2WI (*p* < 0.01).

**Table 2:** Image quality analysis between deep learning reconstructed (DLR) and standard (non-DLR) images based on the consensus review of six readers.

	Median score (DLR)	Median score (non-DLR)	Standardized (Z) test statistic	*p*
PI-QUAL score	4	4	–1.00	0.32
Diagnostic confidence in excluding clinically significant cancer (PI-RADS ≥ 3) in the peripheral zone	4	4	–0.33	0.74
**Diagnostic confidence in excluding clinically significant cancer (PI-RADS ≥ 3) in the transition zone**	**4**	**4**	**–2.31**	**0.02**
**Diagnostic confidence in benign prostatic hyperplasia nodule**	**4**	**4**	**–3.04**	**< 0.01**
**T2-weighted imaging**				
Overall image quality	4	4	–1.265	0.21
**Motion artifacts**	**4**	**4**	**–2.89**	**< 0.01**
Geometric distortion	5	5	0	1.00
Aliasing (wrap-around artifact)	5	5	NA	NA
Metallic artifacts	5	5	1.0	0.32
Zone anatomy	4	4	–0.71	0.48
**Capsule**	**4**	**5**	–**2.45**	**0.01**
Seminal vesicles	4	4	–1.73	0.08
**Ejaculatory ducts**	**4**	**4**	**–2.65**	**0.01**
**Sphincter muscles**	**4**	**4**	**–2.11**	**0.04**
Neurovascular bundles	4	4	0.30	0.76
**Noise**	**4**	**4**	**3.5**	**< 0.01**
**Diffusion weighted imaging (b = 50, 1000, 2000 s/mm^2^) /** a**pparent** d**iffusion** c**oefficient**				
Overall image quality	4	4	0.58	0.56
Motion artifacts	5	5	0.00	1.00
Geometric distortion	5	5	–1.39	0.17
Aliasing (wrap-around artifact)	5	5	–0.63	0.53
Metallic artifacts	5	5	–1.34	0.18
Sharpness	4	4	0.33	0.74
**Noise**	**4**	**4**	**2.11**	**0.04**

Note: Pairwise Wilcoxon signed-rank test with p-value of 0.05 and standardized (Z) test statistic (a positive value indicates that DLR images are superior to standard non-DLR images and, conversely, a negative value indicates that standard non-DLR images are superior to DLR images). Criteria and median scores in bold indicate a statistically significant difference. Considering the pairwise aspect of the analysis, median score can be equal but Wilcoxon signed-rank test significant.

**Fig. 3:**
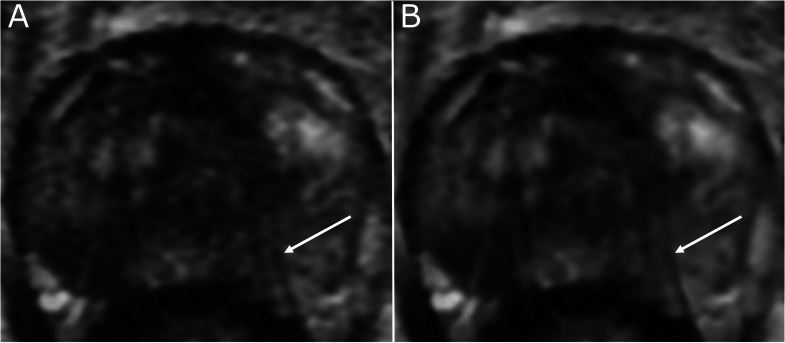
Study is to assess the impact of a commercially available DLR algorithm on prostate MRI: motion artifacts (arrow) on axial T2-weighted images of the prostate, scored 3/5 on standard (non-deep learning reconstructed) images (A), and 2/5 on deep learning reconstructed images (B).

**Fig. 4:**
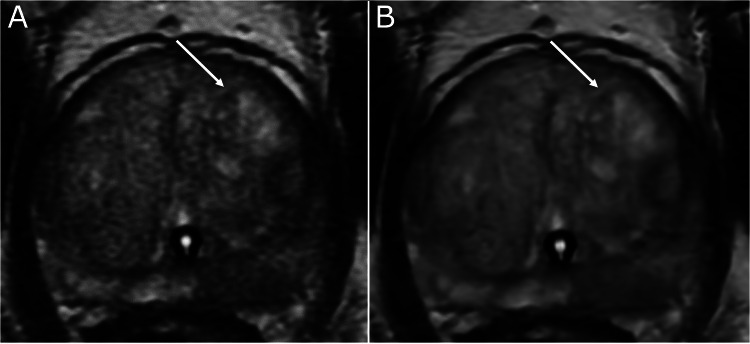
Study is to assess the impact of a commercially available DLR algorithm on prostate MRI: diagnostic confidence and conspicuity of benign prostatic hyperplasia nodule (arrow) on axial T2-weighted images of the prostate, scored 4/5 on standard (non-deep learning reconstructed) images (A), and 3/5 on deep learning reconstructed images (B).

Most image quality features achieved slight agreement among the reviewers (16/23 for DLR images and 14/23 for non-DLR images). Only the assessment of metallic artifacts achieved moderate agreement on both T2WI and DWI sequences considering that only 3 patients with total hip prosthesis replacement were included in the dataset. Consensus was not reached for 1 to 7 features per patient and the additional opinion of the senior radiologist XX was therefore needed (supplemental material - Table S3).Fig. 3, Fig. 4

### 3.3 Radiomics analysis

All segmentations over the patient cohort were subdivided into the following groups: 282 transition zones and 206 peripheral zones. Results for the comparisons between standard non-DLR and DLR images per feature class, prostate zone, and image type are shown (Table 3). The T2WI image pairs resulted in a higher sum of features that differed between the two reconstruction algorithms in both the transition (89 / 93, 96 %) and peripheral zones (91 / 93, 98 %). Conversely, the ADC images had the least amount that differed in the transition zone (55 / 93, 59 %) and the extrapolated b = 2000 s/mm^2^ DWI images had the least amount that differed in the peripheral zone (50 / 93, 54 %). It is noteworthy that the latter two are calculated (synthetic) images. The use of DLR on these parametric maps may have been mitigated in part by image pre-processing steps such as spatial smoothing may be applied prior to ADC calculations or extrapolated b-values.

**Table 3:** Study is to assess the impact of a commercially available DLR algorithm on prostate MRI: number of significantly different features (*p* < 0.05; pairwise Wilcoxon signed-rank test) between deep learning reconstructed (DLR) and standard (non-DLR) images according to Pyradiomics feature class, prostate zone, and image type.

	Sum / Total						
Feature class	First order	GLCM	GLDM	GLRLM	GLSZM	NGTDM	All
T2-weighted imaging							
Transition zone	18 / 18 (100 %)	23 / 24 (96 %)	14 / 14 (100 %)	16 / 16 (100 %)	13 / 16 (81 %)	5 / 5 (100 %)	89 / 93 (96 %)
Peripheral zone	16 / 18 (89 %)	24 / 24 (100 %)	14 / 14 (100 %)	16 / 16 (100 %)	16 / 16 (100 %)	5 / 5 (100 %)	91 / 93 (98 %)
Diffusion-weighted imaging (b = 50 s/mm^2^)							
Transition zone	14 / 18 (78 %)	19 / 24 (79 %)	10 / 14 (71 %)	6 / 16 (38 %)	11 / 16 (69 %)	5 / 5 (100 %)	65 / 93 (70 %)
Peripheral zone	14 / 18 (78 %)	20 / 24 (83 %)	8 / 14 (57 %)	7 / 16 (44 %)	11 / 16 (69 %)	5 / 5 (100 %)	65 / 93 (70 %)
Diffusion-weighted imaging (b = 1000 s/mm^2^)							
Transition zone	18 / 18 (100 %)	17 / 24 (71 %)	9 / 14 (64 %)	12 / 16 (75 %)	7 / 16 (44 %)	4 / 5 (80 %)	67 / 93 (72 %)
Peripheral zone	18 / 18 (100 %)	20 / 24 (83 %)	11 / 14 (79 %)	11 / 16 (69 %)	13 / 16 (81 %)	5 / 5 (100 %)	78 / 93 (84 %)
Diffusion-weighted imaging (extrapolated b = 2000 s/mm^2^)							
Transition zone	12 / 18 (67 %)	12 / 24 (50 %)	11 / 14 (79 %)	13 / 16 (81 %)	11 / 16 (69 %)	3 / 5 (60 %)	62 / 93 (67 %)
Peripheral zone	14 / 18 (78 %)	15 / 24 (63 %)	9 / 14 (64 %)	6 / 16 (38 %)	1 / 16 (6.2 %)	0 / 5 (0 %)	45 / 93 (48 %)
Apparent diffusion coefficient							
Transition Zone	13 / 18 (72 %)	20 / 24 (83 %)	4 / 14 (29 %)	3 / 16 (19 %)	3 / 16 (19 %)	4 / 5 (80 %)	47 / 93 (50 %)
Peripheral Zone	17 / 18 (94 %)	20 / 24 (83 %)	9 / 14 (64 %)	10 /16 (63 %)	9 / 16 (56 %)	5 / 5 (100 %)	70 / 93 (75 %)

GLCM: gray level co-occurrence matrix; GLDM: gray level dependence matrix; GLRLM: gray level run length matrix; GLSZM: gray level size zone matrix; NGTDM: neighbouring gray tone difference matrix.

## 4 Discussion

This study investigated the impact of a commercially available DLR algorithm on prostate MRI images including diagnostic confidence, image quality, and severity of imaging artifacts and radiomics analysis compared to current standard non-DLR reconstruction techniques. Although DLR algorithm reduced the level of noise, it did not improve the overall image quality. It negatively impacted the severity of motion artifacts and degraded anatomical conspicuity and diagnostic confidence in excluding clinically significant cancers (PI-RADS ≥ 3) in the transition zone. Regarding radiomics features, DLR algorithm extensively impacted their values both on prostate T2WI and DWI images.

In the current context of development of artificial intelligence models, these results should call for caution in the use of DLR and underline the need for archiving non-DLR images to the picture archiving and communication systems (PACS) [11], [20], [21]. The significant differences in radiomics features between DLR and non-DLR images suggest that machine learning models previously trained with non-DLR images may not generalize to DLR images. Additionally, as commercial DLR algorithms find wider clinical utility, future machine learning models trained on DLR images may also suffer when retrospectively applied to non-DLR images. Considering that every vendor-specific DLR algorithm is different, further introducing potential heterogeneity to any datasets, these results should strongly encourage research teams to develop machine learning models on non-DLR images. Currently, DLR is the default mode on most commercial MRI scanners. Although safe clinically, DLR should not become the only accessible mode and non-DLR images should remain available both for AI algorithm generation, validation and testing in the research setting and for implementation of regulatory-approved AI algorithms in the clinic. However, even if the non-DLR images are archived in the PACS along with the DLR counterparts, the non-DLR images in this setting may be quite different from the images used to train prior validated machine learning models. For example, when DLR is applied with the main objective to shorten MRI scan time, the non-DLR images will suffer from decreased SNR due to time-saving modifications introduced within the MRI parameters such as fewer signal averages.

As expected, the DLR algorithm reduced the level of noise and smoothed the images without introducing additional blurring [8]. However, the worsening of motion artifacts on the DLR images was not initially anticipated. We hypothesize that the decrease in the level of noise made the images sharper and therefore resulted in the motion artifacts at the edges being more conspicuous. The impact on motion artifacts likely contributed to the decrease in anatomical structural conspicuity and diagnostic confidence in excluding clinically significant cancers (PI-RADS ≥ 3) in the transition zone where the precise delineation of benign prostatic hyperplasia nodules is critical. Altogether, the DLR algorithm did not impact the overall image quality and PI-QUAL score of standard-of-care MRI sequences, which is consistent with prior publications [8,22]. However, these prior studies suggested that DLR could be useful to reduce the acquisition time without compromising image quality.

The main limitation of this study is the size of the dataset. However, these preliminary results raise concerns regarding the impact on the severity of artifacts which could ultimately degrade the overall image quality. Furthermore, the main strength of this study is to raise awareness to the scientific community regarding the deleterious effect of DLR images on the generalizability of machine learning models. The majority of radiomics features were significantly different between DLR and non-DLR images. Consequently, the application of machine learning models to DLR images, that have been trained and tested on standard non-DLR images, would result in erroneous outcome predictions. The robustness of the methodology in this study is that the type of reconstruction of the images (DLR versus standard non-DLR) was the only difference between the two sets of images. The other acquisition parameters including field-of-view, matrix, or number of averages were identical given it was the same acquisition data. The poor inter-reader agreement is a limitation of the study and can be partly explained by the limited size of the dataset. However, the large number of readers (six), the use of a consensus read and the additional opinion of an expert radiologist in discordant cases mitigated this limitation. It is important to emphasize that quality criteria are highly subjective, despite a training session for all readers. In addition, although DLR and non-DLR images were randomized, it remains easy for readers to recognise the type of reconstruction algorithm which could represent a reading bias.

## 5 Conclusion

DLR algorithms perform as an image denoiser without sacrificing sharpness, and preserve overall image quality, but could ultimately worsen motion artifacts and diagnostic confidence in excluding clinically significant cancers (PI-RADS ≥ 3) in the transition zone in significantly motion-degraded MRI examination. Furthermore, the DLR algorithm had a considerable impact on radiomics features both on clinical prostate T2WI and DWI images, which should strongly encourage caution in the sole reliance on DLR images and advocate archiving non-DLR images for both clinical and research purposes.

## Funding

This research did not receive any specific grant from funding agencies in the public, commercial, or not-for-profit sectors.

## Institution review board approval

This retrospective, single-centre study was approved by the institutional Research Ethics Board (2023-9439). Informed consent requirement was waived.

## Research data

Research data are available on demands (Dr Jérémy Dana).

## Declaration of competing interest

The authors declare that they have no known competing financial interests or personal relationships that could have appeared to influence the work reported in this paper.
